# Facial conformation characteristics in Persian and Exotic Shorthair cats

**DOI:** 10.1177/1098612X21997631

**Published:** 2021-03-03

**Authors:** Kerstin L Anagrius, Maria Dimopoulou, Anna N Moe, Ann Petterson, Ingrid Ljungvall

**Affiliations:** 1 University Animal Hospital, Swedish University of Agricultural Sciences, Uppsala, Sweden; 2 Department of Clinical Sciences, Swedish University of Agricultural Sciences, Uppsala, Sweden; 3 Anicura, Roslagen Animal Clinic, Norrtälje, Sweden

**Keywords:** Nose, face, entropion, respiratory sounds, health status, lacrimal apparatus disease

## Abstract

**Objectives:**

The primary objectives of the study were to examine the diversity in facial conformation characteristics within a group of Persian (PER) and Exotic Shorthair (EXO) show cats, and to contrast the results to findings within a group of non-purebred domestic shorthair (DSH) and domestic longhair (DLH) cats. The secondary objectives were to determine the PER/EXO show cat owners’ perceptions of the breathing status of their cats, and to evaluate if remarks from the cat show judges concerning the cats’ head and facial conformation were exclusively related to the aesthetic features of the cats.

**Methods:**

Sixty-four PER and 12 EXO show cats were prospectively examined at five international cat shows, and 20 DSH/DLH cats were examined at an animal hospital. Facial conformation characteristics were evaluated by examining photos of the cats. Owners of the PER/EXO show cats answered a questionnaire concerning their cats’ health status, and they were encouraged to send in the judges’ score sheets from the cat shows.

**Results:**

The PER/EXO show cats had higher diversity in facial conformation characteristics than the DSH/DLH cats, and high incidences of hypoplasia of the nose leather (95%), the nose leather top positioned above the level of the lower eyelid (93%), moderate-to-severe stenotic nares (86%), epiphora (83%) and entropion (32%). Owners of 6/76 PER/EXO show cats stated that their cat had increased respiratory sounds and/or trouble breathing at least once a week. The cat show judges’ written comments were exclusively related to aesthetic features of the cats’ head and facial conformation details.

**Conclusions and relevance:**

Hypoplasia of the nose leather, high position of the nose leather top, stenotic nares, epiphora and entropion were common findings in the PER/EXO show cats but not in the DSH/DLH cats. Few of the cat owners perceived that their cat had problems related to the airways.

## Introduction

The cat has been domesticated for a long time, with data suggesting that the cat has been a highly valued family member for more than 9000 years.^1^ In the process of domestication, active selection of breeding animals has resulted in a variety of hair coats and colours, as well as different conformities of the head, resulting in dolichocephalic, mesocephalic and brachycephalic cat breeds. Brachycephalic cat and dog breeds are characterised by their rounded skull, dorsorotation of the jaws, particularly the maxilla, and a drastic shortening of the nose and face.^2–4^ The brachycephalic conformation has also been shown to be associated with various malformations in the respiratory tract, such as stenotic nares, elongated soft palate (rarely seen in cats) and abnormal nasal turbinates.^2,5–7^ Because of these malformations, secondary complications can also affect the respiratory tract to varying degrees, such as laryngeal oedema, eversion of sacculae and tonsils (rarely seen in cats), and eventual laryngeal collapse.^5,6,8–10^ Brachycephalic airway obstructive syndrome (BOAS) refers to the particular set of upper airway abnormalities that can affect brachycephalic dog and cat breeds. Animals affected can present with signs of heat and exercise intolerance, stertor, stridor, dyspnoea and, in severe cases, cyanosis and syncope.^2,5–7,11^

Most research in the area of brachycephaly and BOAS has been conducted in dogs, but some findings may also be applicable to cats.^7,11,12^ Several studies have shown that an association might exist between brachycephalic features of the face, head and neck, and the existence and severity of BOAS in brachycephalic dog breeds.^5,6,10,13^ A CT-based study evaluating facial bones and head conformation in cats found dorsorotation of the jaw, angulation of the maxillary canine teeth and higher degree of brachycephaly to be associated with more narrow nares, nasal cavity and nasal airways.^3^ A shorter nose has been found to correlate with owner-perceived respiratory noise and breathing difficulties in the cats evaluated.^11^

Organ systems other than the respiratory tract are also affected by the brachycephalic conformation. Brachycephalic cats have been reported to be more prone to eye-related problems such as epiphora,^11^ corneal ulceration^14^ and sequestra, keratitis^15^ and entropion,^16^ dental and coat-related problems,^12^ dystocia^17^ and neurological problems such as an absent menace response, absent postural reactions and ataxia.^18^

Purebred cats are bred according to the breed standard provided by each respective breeders society (breed standards for Persian (PER) and Exotic Shorthair (EXO) cats provided by Fédération Internationale Féline [FIFe; http://www1.fifeweb.org/dnld/std/EXO-PER.pdf] and the World Cat Federation [WCF; http://www.wcf-online.de/WCF-EN/index.html]), and such selection may result in less heterogenicity in specific parts of the genome, thereby giving the specific breed its special appearance.^19^ Cat shows provide the breeders with an opportunity to have their cats compared with the breed standard and other cats of the same breed, and the judges’ assessments are an important factor when breeding animals are selected for future generations. The mating process for the Swedish non-purebred domestic shorthair (DSH) and domestic longhair (DLH) cats is, if even controlled at all, less controlled than for the purebred cats, as the breed standard for the DSH/DLH cats provided by FIFe is less strict and as these cats are often not bred with any standards in mind (breed standard for house cats [DSH/DLH] provided by FIFe, http://www1.fifeweb.org/dnld/std/HCL-HCS.pdf).

The primary objectives of this prospective study were to examine the diversity in facial conformation characteristics within a group of PER/EXO show cats, and to contrast the results to findings within a group of DSH and DLH cats. The secondary objectives were to determine the show cat owners’ perceptions of the breathing status of their cats and to evaluate if the remarks from cat show judges concerning the cats’ head and facial conformation details were exclusively related to aesthetic features of the cats.

## Materials and methods

PER/EXO show cats were prospectively enrolled at five international cat shows in Sweden during 2017 (one cat show arranged by Sveriges Nya Raskattförening (SNRF)/WCF and four arranged by Sveriges Kattklubbars Riksförbund (SVERAK)/FIFe) and 20 DSH/DLH cats were prospectively enrolled at the University Animal Hospital at the Swedish University of Agricultural Sciences in Uppsala, Sweden. Informed owner consent was obtained for all cats participating in the study.

Cats aged ⩾10 months and of both sexes (neutered and intact) were included. PER/EXO show cats had to be declared healthy to participate in shows by an assigned cat show veterinarian (information from these health controls was not used in the present study), and DSH/DLH cats were included if veterinary consultation, at the time of enrolment in the study, was sought for prophylaxis or clinical signs of disease unrelated to respiratory, ophthalmological and odontological conditions.

On the day of enrolment in the study, the owners provided information about their cat, a physical examination of the cat was performed by a veterinarian, photographs were taken of the cat’s head and the show cats were evaluated by the cat show judges.

Information provided by all owners included cat signalment (age, sex, neuter status) and medical history (information about veterinary visits, apart from annual preventive health checks, and also whether or not the cat had undergone upper airway surgery). The owners of the PER/EXO show cats also answered a questionnaire on the same day as their cat was included in the study (Table 1).

**Table 1:** Results from the owner questionnaire

	PER/EXO show cats
Type of food*	Dry: 14 Wet: 2 Both dry and wet: 57
Trouble chewing or abnormal behaviour in feeding situations*	Yes: 1 No: 72
Vomiting/regurgitation*	Seldom or never: 71 Once or several times per month: 1 Once or several times per week: 1 Once or several times per day: 0
Owner’s perception of whether vomiting and/or regurgitation appeared to be problematic for the cat	Yes: 1 No: 68
Epiphora*	Seldom or never: 33 Once or several times per month: 16 Once or several times per week: 16 Once or several times per day: 8
Epiphora staining the fur*	Yes: 25 No: 48
Owner’s perception of whether the presence of epiphora appeared to be problematic for the cat*	Yes: 5 No: 68
Increased respiratory sounds when awake*	Seldom or never: 66 Once or several times per month: 5 Once or several times per week: 1 Once or several times per day: 1
Increased respiratory sounds when asleep	Seldom or never: 66 Once or several times per month: 6 Once or several times per week: 4 Once or several times per day: 0
Increased respiratory sounds or breathing difficulties after activity	Seldom or never: 72 Once or several times per month: 2 Once or several times per week: 2 Once or several times per day: 0
Owner’s perception of whether the presence of respiratory signs appeared to be problematic for the cat	Yes: 3 No: 73

* Results from 73 cats are listed; results from three cats are missing due to incomplete questionnaires

PER = Persian; EXO = Exotic Shorthair

Physical examination of all cats was performed by the same veterinarian (KLA). Examination of the PER/EXO show cats was conducted on a table at the cat shows, whereas examination of the DSH/DLH cats was conducted on a table in an examination room at the animal hospital. All cats were assessed for body condition score (BCS; 1–5-point scale used [the higher score was used if a cat was between scores]), signs of epiphora, entropion and angulation of the maxillary canine tooth (Table 2). Angulation of the maxillary canine tooth was measured with a protractor as the estimated angle between the axis of the canine tooth and a line between the most caudal aspect of crista nuchae and the most dorsal aspect of the nose leather. The left maxillary canine tooth was preferably measured, and the right maxillary canine tooth was only measured if the left was missing. Four different angle categories were established: 66–90°, 46–65°, 26–45° and 0–25° (modified from Schlueter et al).^3^

**Table 2:** Results from the veterinary evaluations

Veterinary examination	DSH/DLH cats	PER/EXO show cats
Physical evaluation		
Entropion	Yes: 0 No: 20	Yes: 24 No: 52
Epiphora	No: 19 Mild: 0 Moderate to severe: 0	No: 13 Mild: 39 Moderate to severe: 24
Angulation of the maxillary canine tooth	66–90°: 13 46–65°: 5 26–45°: 0 0–25°: 0	66–90°: 4 46–65°: 42 26–45°: 23 0–25°: 1
Photographic evaluation		
Type of nares	Rounded: 20 Vertical: 0	Rounded: 28 Vertical: 48
Size of the nose leather	Normal: 18 Hypoplastic: 2	Normal: 4 Hypoplastic: 72
Stenotic nares	No: 20 Mild: 0 Moderate: 0 Severe: 0	No: 1 Mild: 10 Moderate: 27 Severe: 38
Position of the nose leather top	Well below the level of the rima of the lower eyelid: 20 Below the level of the rima of the lower eyelid: 0 At the level of the rima of the lower eyelid: 0 Above the rima of the lower eyelid : 0	Well below the level of the rima of the lower eyelid: 0 Below the level of the rima of the lower eyelid: 0 At the level of the rima of the lower eyelid: 5 Above the rima of the lower eyelid: 71

DSH = domestic shorthair; DLH = domestic longhair; PER = Persian; EXO = Exotic Shorthair

**Figure 1:**
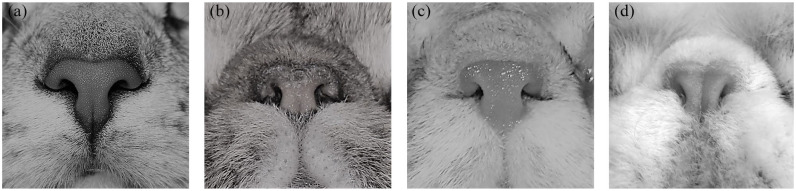
Stenotic nares degree system used in the present study: (a) no stenosis; (b) mild stenosis; (c) moderate stenosis; and (d) severe stenosis

**Figure 2:**
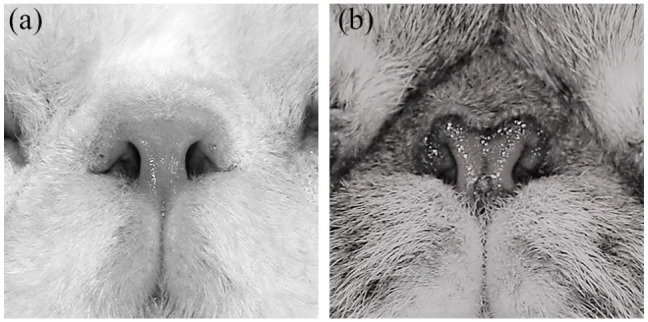
Nose leather size – (a) normal nose leather and (b) hypoplastic nose leather – and type of nares – (a) rounded nares and (b) vertical nares

**Figure 3:**
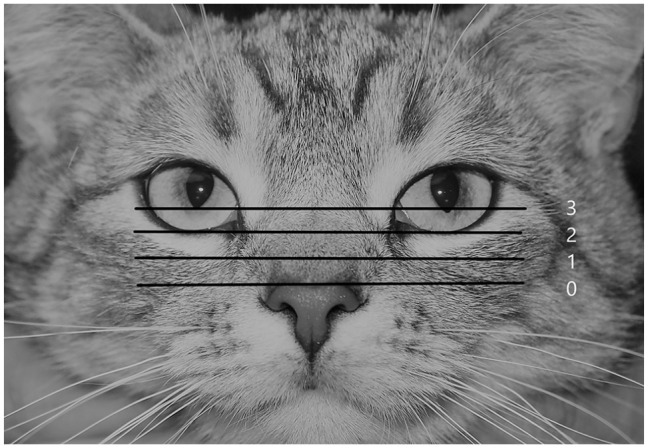
How the position of the top of the nose leather was evaluated in the present study: (0) well below the level of the rima of the lower eyelid; (1) below the level of the rima of the lower eyelid; (2) at the level of the rima of the lower eyelid; (3) above the rima of the lower eyelid

All cats enrolled in the study were photographed in three standardised positions: one straight in front of the cat with its head in a neutral position; one straight in front with the chin slightly elevated; and one with the head in profile. All photographs were taken by the same master thesis veterinary student (ANM), and the positioning of the cats was managed by the same veterinarian (KLA). The photographs were later independently evaluated by two veterinarians (KLA and MD, board certified in surgery) to determine degree of stenotic nares (Figure 1), type of nares (Figure 2), and nose leather size (Figure 2) and position (Figure 3). The inter-observer agreement between the two veterinarians was evaluated and, in cases of disagreement, images were re-evaluated before a final decision was made.

Owners of the PER/EXO show cats were contacted after the cat shows and asked to voluntarily provide the judges’ show score sheets from the day when their cat was enrolled into the study. The score sheets, which contain positive and negative attributes from the judges, were evaluated by one veterinarian (KLA). The score sheets were used only to identify: (1) which face and eye variables had been commented on by the judges; and (2) if the comments provided were exclusively related to the aesthetic features of the show cats’ head/facial conformation details. The described face and eye variables evaluated were compared with information available in the established breed standards for PER/EXO cats (Table 3).

**Table 3:** Overview of head and facial conformation characteristics according to the breed standards of Persian and Exotic Shorthair cats established by the Fédération Internationale Féline (FIFe) and World Cat Federation (WCF), and the number of cats that had received comments on the score sheets by the judges of the cat shows for the various characteristics

Head and facial conformation characteristics described in the breed standards	Summary of the established breed standards	Number of cats assessed for the various head and facial conformational characteristics described in the breed standard*
Shape	Round and massive, well-balanced head, very broad skull	21
Forehead	Rounded forehead	26
Cheeks	Full cheeks	6
Nose	Short and broad but not a snub nose	27
Nose stop	Well-defined definitive stop Stop to be between the eyes, neither above the upper eyelid nor below the lower eyelid^†^	21
Nose leather	Nose leather must be wide, nostrils well open, allowing free and easy passage of air^†^ The upper edge of the nose leather is not higher than the lower eyelid^‡^	2
Chin	Strong chin	18
Jaws	Broad and massive jaws	7
Expression	Nice open and expressive face	2
Eye shape	Round and open eyes	22
Eye size	Large eyes	21
Eye placement	Eyes set wide apart	3
Eye colour	Brilliant and expressive, pure colour corresponding with the colour of the coat	29

* Results from 31 cats are listed

† Only in the breed standard provided by FIFe

‡ Only in the breed standard provided by WCF

### Statistical analysis

Data were analysed using commercially available statistical software (JMP Pro version 14.0.0; SAS Institute). Data are presented as descriptive statistics. Spearman’s rank-order correlation analyses were performed to evaluate the nature of potential associations existing between different variables obtained at the physical examination (epiphora, entropion, angulation of the maxillary canine tooth) and from the photographs of the cats’ heads (degree of stenotic nares, type of nares, and nose leather size and position). Level of significance was set at *P* <0.05.

## Results

### Cats

A total of 76 PER/EXO show cats (64 PER and 12 EXO) were included in the study, of which 42 were females (13 neutered and 29 intact cats) and 34 were males (14 neutered and 20 intact cats). All of the PER/EXO show cats had a typical brachycephalic appearance. Twenty DSH/DLH cats, of which eight were females (all neutered) and 12 were males (11 neutered and one intact), were included. All of the DSH/DLH cats had a typical mesocephalic appearance. The PER/EXO show cats had a median age of 3.1 years (interquartile range [IQR] 1.7–4.9 years), and the DSH/DLH cats had a median age of 6.7 years (IQR 3.3–11.9 years). Of the PER/EXO cats, 50 had a BCS of 3, 17 had a BCS of 4 and one had a BCS of 5. Of the DSH/DLH cats, one had a BCS of 2, 10 had a BCS of 3, four had a BCS of 4 and one had a BCS of 5. Information about the BCS was missing for eight PER/EXO cats and for four DSH/DLH cats. Previous dental problems were reported by the owners of 3/76 PER/EXO cats and 4/20 DSH/DLH cats; previous ophthalmological problems were reported by the owners of 5/76 PER/EXO and 1/20 DSH/DLH cats. None of the cats was reported by their owner to have previously undergone upper airway surgery.

### Owner questionnaire

Results from the questionnaire from the PER/EXO show cat group are listed in Table 1. All cats included were living in multi-cat households (median number of cats 6; IQR 4–9), and all cats lived with the person attending the cat show.

Owners of 8% of cats stated that their cats had increased respiratory sounds and/or trouble breathing at least once a week, and owners of 4% of all cats answered that they found their cat to have airway-related problems.

### Physical veterinary examination

Epiphora was seen in 83% of the PER/EXO show cats, whereas epiphora was not seen in any of the DSH/DLH cats (Table 2). Entropion was seen in 32% of the PER/EXO show cats, and was located medially on the lower eyelid in all of these cats (Table 2). Entropion was not seen in any of the DSH/DLH cats.

Angulation of the maxillary canine tooth could be clearly estimated in 70/76 PER/EXO show cats (Table 2). Two PER/EXO show cats lacked the left maxillary canine and the right maxillary canine was therefore measured. Because of lack of compliance in five cats, it could not be ascertained if the angulation of the maxillary tooth was in the range of 26–45° or 46–65° in four cats, or in the range of 0–25°or 26–45° in one cat. One PER/EXO show cat lacked both maxillary canine teeth. Angulation of the canine tooth could be estimated in 18/20 DSH/DLH cats; 2/20 DSH/DLH cats lacked both maxillary canine teeth (Table 2).

### Evaluation of the photographs

The profile photographs were excluded from the final evaluation because it was difficult to identify facial details of interest in a standardised way in most PER/EXO show cats in these photographs. In the photographs taken in front of the cats, the facial details could be identified and evaluated in all cats. An initial inter-observer agreement between the two veterinarians was found for 364/384 (95%) observations assessed on the photographs, and a final agreement was reached after discussion between the two veterinarians for the rest of the variables.

The kappa coefficient for the agreement between the two veterinarians ranged between 0.86 and 0.98 for the four facial variables investigated.

The PER/EXO show cats showed higher within-group diversity in the assessed facial conformation details compared with the DSH/DLH cats (Table 2).

Moderate-to-severe stenotic nares were seen in 86% of the PER/EXO show cats, whereas stenotic nares were not identified in any of the DSH/DLH cats. Hypoplastic nose leather was seen in 95% of the PER/EXO show cats, whereas all of the DSH/DLH cats had nose leather of a normal size. The position of the top of the nose leather was above the level of the rima of the lower eyelid in 93% of the PER/EXO show cats, whereas all of the DSH/DLH cats had a nose positioned well below the level of the rima of the lower eyelid.

### Spearman’s rank-order correlations

Spearman’s rank-order correlation analyses were not performed for the DSH/DLH cats owing to low within-group diversity for variables analysed for this group of cats (Table 2). The type and position of the nose leather were not analysed for the PER/EXO show cats due to low within-group diversity for these variables.

Entropion was found to be associated with epiphora (*P* <0.0049) and vertical nares was associated with more severe stenotic nares (*P* <0.0001).

### Evaluation of the cat show score sheets

Score sheets from the judges of cat shows were received from owners of 31/76 cats. Variables of the head assessed by the judges are presented in Table 3. The majority (85%) of the 232 comments regarding the head and eyes were positive and included comments such as ‘excellent deep stop’ and ‘excellent small nose’. Negative comments (10%) included information such as ‘nose a little long’ and ‘head not rounded enough’. Five percent of the comments could not be clearly interpreted to be of positive or negative character, or could not be evaluated owing to difficulties interpreting the handwriting. The evaluated comments provided by the judges on the score sheets were exclusively related to aesthetic features of the show cats’ head/facial conformation details. None of the cats diagnosed with epiphora, entropion and/or stenosis of the nares at the physical veterinary examination at the cat shows, and from evaluation of the photographs taken at the cat shows, received any comments about these findings in the judges’ score sheets.

## Discussion

In the present study, the PER/EXO show cats showed more diversity in facial conformation characteristics than the DSH/DLH cats. A majority of the PER/EXO show cats presented with stenotic nares, hypoplasia of the nose leather and epiphora. Approximately one-third of the PER/EXO show cats presented with entropion of the lower eyelid and >90% of the PER/EXO show cats presented with their nose leather top positioned above the rima of the lower eyelid.

Moderate-to-severe stenotic nares were found in 86% of the PER/EXO show cats, and more severe stenotic nares were associated with vertical nares. A similar association has previously also been reported in dogs.^9,13^ Furthermore, stenotic nares were found to be associated with hypoplasia of the nose leather in the PER/EXO show cat group. The risk of developing BOAS has been shown to be higher in dogs with more stenotic nares.^10,13,20^ Studies evaluating the potential correlations between exterior facial conformation characteristics and functional BOAS score in brachycephalic cats (eg, the exercise tolerance test), have – to our knowledge – never been published. The functional BOAS assessment was not performed in the present study. A number of variables were identified at the veterinary examination that could potentially indicate problems related to the airways. Despite this, owners of 6/76 PER/EXO show cats stated in the questionnaire that their cat had increased respiratory sounds and/or trouble breathing at least once a week, and only three owners perceived their cat to have problems related to the airways. Cats with a brachycephalic appearance have previously been reported to present with increased respiratory sounds.^11^ Previous studies have also suggested that signs of disease, especially if frequently seen in animals of particular breeds, may be normalised among their owners. This, together with the fact that veterinarians are trained at identifying and recognising these signs, possibly explains the difference in perception of problematic airway-related signs assessed by owners and veterinarians.^21–23^

Although 80% of PER/EXO show cats were assessed by the veterinarian of the study to have epiphora, only 33% of cat owners reported that their cat had epiphora at least once weekly. This discrepancy may again, potentially, be explained by normalisation among owners to typical features commonly seen in cats of these breeds. The owners may also, potentially, have difficulty recognising signs of disease.^23^ In addition, the assessment of epiphora was performed in different settings (home vs cat shows), which could have affected the results.

Entropion was significantly associated with epiphora in the PER/EXO show cat group (*P* <0.0049), and a similar association has previously been described in dogs.^24^ Brachycephalic cats have previously been found to be predisposed to corneal sequestra^15^ and entropion,^16,25^ and brachycephalic cats have, furthermore, been shown to have a decreased corneal sensitivity vs DSH cats.^26^ The brachycephalic conformation has also been found to affect the nasolacrimal duct and reduce the nasolacrimal drainage because of foreshortening of the face and dorso-rotation of the maxilla and maxillary canine tooth.^3,4^ All of these factors can potentially be involved in development of epiphora in cats,^3,4,15,16,25^ and, therefore, explain the high incidence of epiphora among the PER/EXO cats.

The angulation of the maxillary canine teeth was not statistically associated with any of the facial variables investigated in the present study. A previous study suggested the angle of the maxillary canine tooth to be a good indicator of the degree of brachycephaly in cats.^3^ The degree of brachycephaly was not investigated in our study, but the findings from the above-mentioned study^3^ and the present study indicate that the angulation of the maxillary canine may be linked to the conformation of the cranium rather than to facial conformation characteristics in brachycephalic cats.

All DSH/DLH cats had a mesocephalic appearance, and the low within-group diversity in facial conformation characteristics found among the DSH/DLH cats is an interesting finding as the mating process of Swedish DSH/DLH cats is not commonly planned by the owners. Furthermore, the breed standard for DSH/DLH cats (also named house cats in the breed standard) provided by the FIFe is less strict than for purebred cats, and few non-purebred cats participate in cat shows. Many DSH/DLH cats in Sweden have daily outdoors access, and even if the vast majority are neutered (https://jordbruksverket.se/download/18.514d3694172cce072377578b/1592688102970/Hundar%20och%20katter.pdf), non-neutered cats may also be allowed outdoors and may therefore mate in an unselective manner. The breeding of show cats is usually more carefully planned, and published breed standards, judges’ assessments and scores at cat shows are all likely to have an impact on the selection of breeding animals. Yet, compared with DSH/DLH cats, the higher diversity in facial conformation characteristics for the PER/EXO cats could indicate that personal preferences in desirable characteristics exist among the breeders, and potentially also the cat show judges.

To our knowledge, no previously published studies have assessed cat show judges’ assessments of cats’ facial characteristics based on available information provided on the score sheets from cat shows. A relatively limited number of score sheets were evaluated in the present study, but the results provide an insight into how cats are judged at cat shows. According to the breed standards provided by the FIFe and WCF, 25/30 points out of a maximum score of 100 points are assigned to variables related to the head, and a maximum of 15 points are assigned to the eye colour of the cat. The comparably high number of points assigned to the head and eye colour indicates that these features are important when selecting desired appearances. All judges commented on variables regarding both the head and eyes, but the comments were exclusively related to aesthetic features of the conformation characteristics. None of the cats was described to have signs of epiphora, entropion or stenotic nares based on the available information in the score sheets from the judges. Both entropion and ‘narrow nostrils’, which are considered ‘general faults’ for all breeds at cat shows (http://www1.fifeweb.org/dnld/std/GEN-FAULTS.pdf), were noted in a high number of cats during the veterinary examination performed in the study and on the photographs taken at the cat shows. Whether all aspects of the established breed standards regarding head and eyes were assessed in the individual PER/EXO show cats could not be evaluated based upon the information described in the score sheets.

The comments from the judges were very diverse concerning terminology and content, which made a structural compilation hard to accomplish. More standardised score sheets, which more strictly follow the information described in the published breed standards, could potentially ease the interpretation of the score sheets and, furthermore, strengthen the health perspectives of the breed descriptions. The criteria used in the score sheets could also be exemplified with images. Such an approach could thereby facilitate selection of optimal cats for future breeding.

### Study limitations

The DSH/DLH cats were enrolled at a time when their owners were seeking veterinary consultation for their cat, and all cats were therefore not declared as clinically healthy when enrolled into the study. The DSH/DLH cats were, however, included for within-group comparison of facial conformation characteristics with findings from the PER/EXO show cat group, and their general health status was therefore not considered mandatory. The comparably low number of DSH/DLH vs PER/EXO show cats is also a potential limitation. However, the DSH/DLH cats were primarily included for comparison with the PER/EXO show cats and the DSH/DLH cats were therefore not the main focus of the present study.

Two cat breeds were enrolled into the PER/EXO show cat group, but the results from these breeds were not separated. These two are sister breeds and are, except from the haircoat, bred towards the same breed standards, and cats of these breeds are in general derived from the same ancestors (https://cfa.org/exotic/exotic-article/) and can be bred together without restrictions.

Examination of the score sheets for the cats that had participated at cat shows were not included in the original study plan, but it turned out to be an important addition to the study. The owners were contacted after the cat shows to voluntarily submit assessments and the response rate might have been higher if this strategy had been included in the original study plan.

## Conclusions

PER/EXO show cats showed higher diversity in facial conformation characteristics than the DSH/DLH cats. Moderate-to-severe stenotic nares and presence of epiphora were observed in over 80% of the PER/EXO show cats, but few of the PER/EXO show cat owners perceived their cat to have problems related to the airways or problems related to presence of epiphora. Approximately 30% of the PER/EXO show cats presented with entropion of the lower eyelid. Comments provided by the judges at the cat shows about the cats’ head and facial conformation details were exclusively related to aesthetic features of the cats.

Potential correlations between exterior facial conformation characteristics and functional BOAS score should be evaluated in future studies.
